# Optimization of a Method to Isolate and Culture Adult Porcine, Rats and Mice Müller Glia in Order to Study Retinal Diseases

**DOI:** 10.3389/fncel.2020.00007

**Published:** 2020-01-29

**Authors:** Xandra Pereiro, Noelia Ruzafa, Arantxa Acera, Aritz Urcola, Elena Vecino

**Affiliations:** ^1^Experimental Ophthalmo-Biology Group, Department of Cell Biology and Histology, University of the Basque Country UPV/EHU, Leioa, Spain; ^2^Department of Ophthalmology, University Hospital of Alava, Vitoria-Gasteiz, Spain

**Keywords:** cell culture (cell cultivation), glia, retina, neuroprotection, methods

## Abstract

Müller cells are the predominant glial elements in the retina, extending vertically across this structure, and they fulfill a wealth support roles that are critical for neurons. Alterations to the behavior and phenotype of Müller glia are often seen in animal models of retinal degeneration and in retinal tissue from patients with a variety of retinal disorders. Thus, elucidating the mechanisms underlying the development of retinal diseases would help better understand the cellular processes involved in such pathological changes. Studies into Müller cell activity *in vitro* have been hindered by the difficulty in obtaining pure cell populations and the tendency of these cells to rapidly differentiate in culture. Most protocols currently used to isolate Müller glia use neonatal or embryonic tissue but here, we report an optimized protocol that facilitates the reliable and straightforward isolation and culture of Müller cells from adult pigs, rats and mice. The protocol described here provides an efficient method for the rapid isolation of adult mammalian Müller cells, which represents a reliable platform to study therapeutic targets and to test the effects of drugs that might combat retinal diseases.

## Introduction

The Müller cell is the predominant glial cell in the retina, representing 90% of the retinal glia. These cells are radially oriented cells that traverse the retina from its inner border to the distal end of the outer nuclear layer. They occupy an important position in the retina due to their role in connecting neurons and nerve fibers (Reichenbach and Robinson, 1995; Bringmann et al., 2006). Müller cells are born in the late stages of retinal histogenesis, when the majority of neuronal cell types have already been generated. This enables the Müller cells to establish an anatomical and functional link between retinal neurons and the compartments with which they exchange molecules, such as retinal blood vessels, the vitreous chamber and the sub-retinal space (Vecino et al., 2016).

The importance of Müller cells in the retina is such that in the last 20 years, research into these cells has increased substantially and they are now known to fulfill many crucial roles in the retina. Müller cells provide neurons with trophic substances and they remove metabolic waste, also playing a critical role in regulating the volume of the extracellular space, as well as maintaining ion and water homeostasis of the retina, and the blood-retinal barrier. They even release gliotransmitters and other neuroactive substances, regulating synaptic activity through neurotransmitter uptake and recycling, and providing neurons with neurotransmitter precursors. Müller cells are also important structural elements in the retina, buffering mechanical deformations in the tissue, and they guide light to the photoreceptors and (Sarthy and Ripps, 2001; Reichenbach and Bringmann, 2010). These cells modulate immune and inflammatory responses, and they are the first cells to respond to retinal insult, becoming rapidly activated in order to safeguard the retinal structure and immune privileges in the nervous tissue (Ash et al., 2014). Moreover, in response to infection and certain inflammatory scenarios, Müller cells undergo reactive gliosis. In addition, Müller glia offer neuroprotection to RGCs (Garcia et al., 2002; Ruzafa and Vecino, 2015), facilitating their sprouting and neurite regeneration through the secretion of specific factors (Ruzafa et al., 2018). They can also activate and protect photoreceptors (Del Rio et al., 2011), and if they cease to support retinal neurons, they contribute to neurodegeneration (Bringmann and Wiedemann, 2012; Pereiro et al., 2018). Müller cells are also considered to represent a potential source of adult stem/progenitor cells thus, they will have a major impact to future cell-based therapeutic approaches in retinal degenerative diseases (Reichenbach and Bringmann, 2013; Vecino et al., 2016).

Although they are implicated in retinal diseases (e.g., glaucoma, diabetic retinopathy, age-related macular degeneration, retinitis pigmentosa, etc.) and neural regeneration, the heterogeneity between individual Müller cell types is not well understood. Much of the current information about Müller cells, their activity and dysfunction has been derived from animal models, and mainly from cultured Müller cells. However, while there are several protocols for the isolation of Müller cells from different mammalian species, these generally rely on the use of tissue from neonatal animals to get primary cell cultures (Table 1), probably due to the loss of regeneration potential as mammals age (Fischer et al., 2002; Schafer and Karl, 2017). However, degenerative retinal diseases are more frequent in adults and consequently, *in vitro* model systems developed with adult cells would be more useful to investigate the physiological and pathological events that occur in the mature retina. Until recently, certain problems associated with primary Müller cell cultures limited their utility to study these cell’s functions *in vitro*, not least the difficulty in obtaining pure cell populations free of contamination by astrocytes, microglia or neurons. Consequently, the majority of studies focusing on these cells have used permanent cell lines obtained through the immortalization of primary cells with viral oncogenes. Various Müller cell lines have been reported in the literature, including the rMC-1 Müller cell line from the adult rat retina (Sarthy et al., 1998), the first permanent Müller cell line, as well as the TR-MUL cell line (Tomi et al., 2003), the spontaneously immortalized human Müller cell line (MIO-M1) (Limb et al., 2002) and novel spontaneously immortalized rat Müller cell line, SIRMu-1 (Kittipassorn et al., 2019). Cell lines can change genetically over multiple passages, leading to phenotypic differences that leads to potentially different experimental results. Indeed, the transformation process can alter other characteristics in addition to proliferation and life span compared to primary cells, such as cellular, metabolism (Bissell et al., 1972) and epigenetic changes (Simboeck et al., 2011). Thereby, cell lines can lose tissue-specific functions and acquire a molecular phenotype quite different from cells *in vivo* (Pan et al., 2009). For Example, the MIO-M1 line might exhibit progenitor characteristics, it may also express markers of post-mitotic retinal neurons like opsines (Hollborn et al., 2011). Hence, primary cultures are considered by many researchers to be more physiologically similar to the cells *in vivo*, thereby representing a more suitable experimental model to reflect the *in vivo* state.

**TABLE 1:** Comparison of the Müller cells culture methods published for mammals.

Species	Animal’s age	Retina removed after digestion	Digestion Protocol	Substrate	Medium	Change of medium	References
Rabbit	N/S	No	Collagenase (4 mg/ml) + hyaluronidase 200 U/ml) + medium containing Ca^2+^/Mg^2+^ 20′ + papain (26.4 U/ml, pH 6.5) 10′	N/S	N/S	N/S	Trachtenberg and Packey, 1983
Cat	Adult	No	Ca^2+^/Mg^2+^-free BSS + 0.5 mg ml Nagarse (Protease type XXVII) 37°C, 30′.	0.1 mg/ml Poly-L-lysine.	DMEM with FCS	Every 5 days	Lewis et al., 1988
Rabbit	P6	No	0.125% trypsin/0.05% DNase room temperature 10′ and then 37°C, 10′.	Poly-L-lysine (0.1 mg/ml) coated glass-coverslips	DMEM with FCS	N/S	Scherer and Schnitzer, 1989
Rat	P8-12	Yes	DMEM + 0.1% trypsin +70 U mL^–^collagenase, 37°C for 60′	Untreated sterile glass coverslips	DMEM with 2 mM glutamine	After the first 6 days, replenished every 3-4 days	Hicks and Courtois, 1990
Human	19–88 years	No	CMF + 0.1% trypsin + 0,2% hyaluronidase + 4% chicken serum, 37°C, 45′	N/S	DMEM and Ham′s F12 with FBS	Twice weekly	Puro, 1991
Rabbit	P3	No	Ca^2+^ free solution + 0.5 mg/ml papain, 35°C, 35′	N/S	DMEM with 10% FBS	N/S	Reichenbach et al., 1995
Pig	N/S	No	10 ml L15 + 17 U/ml papain, 34°C, 60′ and L15 + 150 U/ml DNase, 34°C, 30′ and Percoll density gradient.	0.1 mg/ml monomeric type I Collagen	DMEM containing 20 mM Hepes and 10% FBS	N/S	Guidry, 1996)
Rabbit	N/S	No	Papain (130 μ/10 ml DMEM) + 1 mM EDTA+ 4.5 mg cysteine, 4°C, 45′	N/S	DMEM with 10% FBS	Every 2–3 days	McGillem et al., 1998
Pig	Adult	No	10 ml L15 + 17 U/ml papain, 34°C, 60′, L15 + 150 U/ml DNase, 34°C, 30′ and Percoll density gradient.	poly-D-lysine (2 μg/cm^2^) and laminin (1 μg/cm2)	DMEM with 10% FBS	N/S	Garcia et al., 2002
Pig	Adult	No	Papain (2.2 U) for 40 min at 37°C and Percoll density gradient.	Plated directly onto cell culture plates (NUNC)	DMEM with 10% FCS	N/S	Hauck et al., 2003
Rat	P8-10	N/S	DMEM + 0.1% trypsin + 70 IU/ml collagenase, 37°C, 30′	N/S	DMEM with 10% FBS	N/S	Lamas et al., 2007
Mouse	P12 and 4 weeks	No	Papain (180 units/mL) + DNase, 37°C, 8–10 min.	N/S	NBA with 10% FBS, 1 mM L−glutamine, 1% N2 and EGF (100 ng/m)	N/S	Ueki et al., 2012
Mouse	P10 or P14	No	Papain for 45 min at 37°C	Poly-D-lysine (50 mg/ml) and laminin (0.5 mg/ml).	DMEM/F12	N/S	Schafer and Karl, 2017

*FBS, fetal bovine serum; FCS, fetal calf serum; N/S, not stated; NBA, neurobasal medium.*

The protocols that currently exist to culture Müller cells offer little insight to allow their successful reproduction. Thus, we describe here a protocol that is fast, easy to reproduce, and that has been optimized for adult tissue from different mammalian species, thereby providing a valuable tool to study diseases involving Müller cells. Different enzymes for digestion, substrates and culture mediums have been compared in order to establish the optimal protocol. Using this culture method for adult Müller cells, their phenotypic characteristics can be readily characterized *in vitro*, including the specific Müller cell markers expressed. In conclusion, the protocol provides a useful method to culture adult mammal Müller cells, a model that can be used to analyze the behavior of these glial cells when subjected to certain stresses *in vitro*, mimicking certain retinal diseases with a view to understanding their etiology.

## Materials and Methods

### Animals

Eyes from 12 months old adult pigs were obtained from a local abattoir and transported to the laboratory in cold CO_2_-independent Dulbecco’s modified Eagle’s medium (DMEM/-CO_2_; Gibco-Life Technologies). Adult 2 month old Sprague Dawley rat and BALB/c mouse eyes were obtained from animals reared at the University’s animal house (University of the Basque Country, UPV/EHU). Animals were kept in standard housing conditions on a 12 h light-dark cycle, with *ad libitum* access to food and water. Rats were humanely sacrificed by exposure to CO_2_ and mice were sacrificed by cervical dislocation. This study was carried out in strict accordance with the recommendations in the Guide for the Care and Use of Laboratory Animals. The experimental protocol met European (2010/63/UE) and Spanish (RD53/2013) standards for the protection of experimental animals, and it was approved by the Ethical Committee for Animal Welfare of the University of Basque country.

### Isolation and Culture of Adult Müller Cells

Pig eyes were dissected within 1–2 h of enucleation, and those of rats and mice immediately after enucleation. The retina was isolated in fresh DMEM/-CO_2_ medium by circumferential section of the cornea and removal of the anterior chamber. Major blood vessels were excised in the case of the pig retina and the retinas were then chopped up into small fragments. In order to establish the best protocol, different enzymes for digestion, substrates and culture media were tested. The retinas were incubated at 37°C for 30 min in (1), a Sterile Earle′s Balanced Salt Solution (EBSS) containing Papain (20 U/mL) and DNase (2000 U/mL: Worthington, Lakewood, NJ, United States), or (2), a Trypsin-EDTA solution (0.25%: Life Technologies, Carlsbad, CA, United States). To stop the enzyme digestion, DMEM containing 10% FBS (fetal bovine serum) was added for 5 min at room temperature, and the tissue was then dissociated mechanically by careful homogenizing with pipettes of different tip sizes, recovering the cells by centrifugation at 1200 rpm for 5 min. The pelleted cells were re-suspended and cultured in three different media: (1) DMEM + 10% FBS; (2) DMEM-F12; and (3) Neurobasal A supplemented with B27 + 10% FBS (Life Technologies, Carlsbad, CA, United States).

The cells were seeded onto sterile 12 mm glass coverslips in 24 well plates, coated with (1) poly-L-lysine (100 μg/ml: Sigma-Aldrich, St. Louis, MO, United States) alone or with (2) laminin (10 μg/ml: Sigma-Aldrich, St. Louis, MO, United States), or (3) left untreated. The amount of the cells seeded was: the circumference of a pig retina (8 mm diameter) per 4 wells; 1 rat retina per 8 wells and 1 mouse retina per well.

The cultures were maintained in a humidified incubator at 37°C in an atmosphere of 5% CO_2_, 95% O_2_. The medium was totally replaced with fresh medium on day 1 of culture, by which time the Müller cells were mostly the cell type that had attached to the coverslips and thus, the other cell types were simply washed away with the debris facilitating the pure culture of Müller cells. Subsequently, the culture medium was changed every 3 days by replacing half the volume of the medium with fresh medium. These cultures reached confluence after 7 days *in vitro* (DIV), this time point was stablished as the best for our study, since it has been previously demonstrated a possible transdifferentiation of Müller cells after 14 days *in vitro* (Guidry, 1996; Hauck et al., 2003).

### Immunocytochemistry

After 7 days *in vitro*, the cells were washed in PBS (phosphate buffered saline, pH 7.0), fixed in methanol for 10 min at −20°C and non-specific antigen binding was blocked for 30 min at room temperature with blocking buffer (0.1% Triton X-100 and 3% BSA-bovine serum albumin-in PBS). The primary antibodies used are shown in Table 2.

**TABLE 2:** Primary antibodies used.

Antigen	Host	Dilution	Supplier	Ref.
GFAP	Rabbit	1:1,000	Sigma-Aldrich, St. Louis, MO, United States	G9269
Glutamine Synthetase	Rabbit	1:10,000	Abcam, Cambridge, United Kingdom	Ab49873
p75NTR	Rabbit	1:2,000	Abcam, Cambridge, United Kingdom	Ab8877
Vimentin	Mouse	1:2,000	Dako, Glostrup, Denmark	M0725
CRALBP	Rabbit	1:2000	Abcam, Cambridge, United Kingdom	ab154898
Beta III-Tubulin	Rabbit	1:2000	Abcam, Cambridge, United Kingdom	Ab18207
Iba-1	Rabbit	1:1000	Wako, Richmond, VA, United States	016–20001

The antibodies were diluted in blocking buffer and incubated with the cultures overnight at 4°C. The cells were washed three times with PBS and the cultures were exposed for 1 h at room temperature to the corresponding secondary antibodies at a dilution of 1:1,000 in PBS/BSA (1%): Alexa Fluor 488 and Alexa 555 conjugated goat anti-mouse and goat anti-rabbit antibodies (Invitrogen, Eugene, OR, United States). Following three washes of the cells, the coverslips were mounted with Fluor-save Reagent (Calbiochem, San Diego, CA, United States).

### Flow Cytometry Analysis

After 7 days *in vitro*, the cells were trypsinized and washed with PBS, and recovered by centrifugation at 1200 rpm for 5 min. The cells were resuspended in 1 ml of PBS, fixed and permeabilized for 1 h at 4°C by adding ethanol 100% to a final concentration of 70% ethanol. The cells were then washed and resuspended in PBS + BSA (5 mg/ml) for 30 min at room temperature to block non-specific antigen binding, and they were then stained overnight at 4°C with an anti-p75NTR antibody with shaking (Table 2). After washing for 1 h at room temperature, the primary antibody was detected with a secondary Alexa Fluor 555 conjugated goat anti-rabbit antibody (Invitrogen, Eugene, OR, United States) and the cells were analyzed on a FACScan (Beckman Coulter Gallios, Brea, CA, United States) to identify those positive for p75NTR.

### Flow Cytometry Analysis of Cell Cycle Profiles

Müller cell cultures at 3 and 7 DIV were washed with PBS and 0.02% EDTA, adding 0.025% trypsin for 5 min at 37°C. After centrifugation, the cells were fixed in 70% ethanol and aliquots of 1 × 10^6^ cells were incubated with propidium iodide (10 μg/mL) in presence of RNase A (250 μg/mL) for 1 h at 4°C. The cell cycle was then analyzed on a FACScan (Beckman Coulter Gallios, Brea, CA, United States).

### Time-Lapse Analysis of Müller Cell Cultures

Time-lapse analysis of the proliferation kinetics in pig and rat Müller cell cultures was performed, studying several variables: (1) the time that the same cell takes to divide again, the time between divisions; (2) the time from prophase to cytokinesis, considered as the time that Müller cells take to divide; and (3) the percentage of Müller cells that divide in the same field. We analyzed each field over 8 h as this is the shortest time required for a cell to divide, thereby avoiding counting the same cell twice. The analysis was performed by taking one frame every 10 min, over 72 h from the 3rd to 6th day in culture, using a 20X objective and a Zeiss Axio Observer (Zeiss, Jena, Germany) coupled to a digital camera (Zeiss Axiocam MRM, Zeiss, Jena, Germany) controlled by the Zen software (Zeiss, Jena, Germany). During imaging, the cells were maintained in a PM S1 incubator (Zeiss, Jena, Germany) at 37°C under a humidified atmosphere of 5% CO_2_ in air. At least eight different fields from three coverslips were analyzed for each experimental condition and from three independent experiments on pig, rat and mouse cultures.

### Quantification and Statistical Analysis of Müller Cells

Müller cells were analyzed on an epifluorescence microscope (Zeiss, Jena, Germany) coupled to a digital camera (Zeiss Axiocam MRM, Zeiss, Jena, Germany). A mosaic of the entire coverslip was obtained with a 10X objective and once the mosaic was defined, the coverslip surface area was calculated (132.73 mm^2^). The semi-automatic Zen software (Zeiss, Jena, Germany) was used to count the total number of nuclei stained with DAPI, taking into consideration the limits of the axis of the nuclei of Müller cells to achieve more accurate measurements. As such, we used a specific macro designed to measure the limits of the axes (10–40 μm), which was corrected manually for each image. At least three coverslips were analyzed for each experimental condition and from three independent experiments.

Müller cell density (cells per cm^2^) and the parameters measured in time-lapse analysis were described as the mean and standard error of mean, and these parameters were compared between the different conditions. Statistical analyses were carried out using IBM SPSS Statistics software v.21-0 and the homogeneity of the variances was assayed with Levene’s test. A Mann–Whitney *U* test or ANOVA were used to assess whether there were significant differences between the groups. The minimum value of significance for both tests was defined as *p* < 0.05.

## Results

### Enzymes for Digestion

**FIGURE 1:**
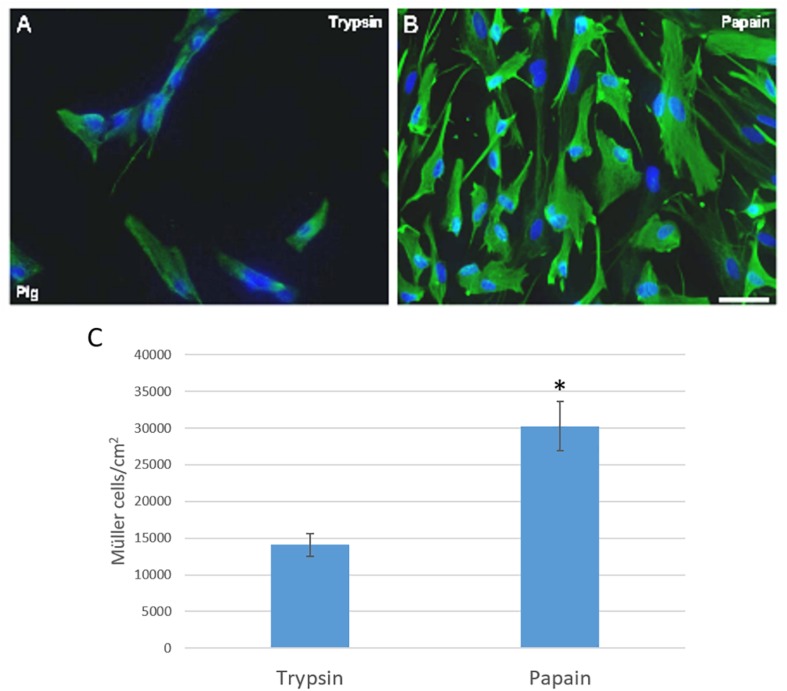
Analysis of pig Müller cell cultures derived from retinas digested with papain or trypsin. Images from Müller cell cultures derived from retinas digested with trypsin **(A)** or papain **(B)**. Müller cells are labeled with an antibody against vimentin (green) and the nuclei are stained with DAPI (blue). The histogram represents the analysis of the number of Müller cells after 7 DIV **(C)**. The survival of Müller cells after 7 DIV increased significantly when the retina is digested with papain: ^∗^*p*-value < 0.05. Scale bar, 100 μm.

In order to establish the best protocol to culture adult mammalian Müller cells, we evaluated different parameters to define the best conditions for Müller cell survival and proliferation. To initially digest the tissue, we evaluated the benefits of using two different enzymes, papain or trypsin. Following digestion of the tissue with either of these enzyme and mechanical dissociation, the total number of Müller cells in culture was analyzed after 7 DIV. Accordingly, the number of viable pig Müller cells in culture was seen to be significantly higher after digesting the retina with papain (30,233.67 ± 6,697.33 Müller cells/cm^2^) than with trypsin (14,083.59 ± 2,635.36 Müller cells/cm^2^: Figure 1).

### Substrates

**FIGURE 2:**
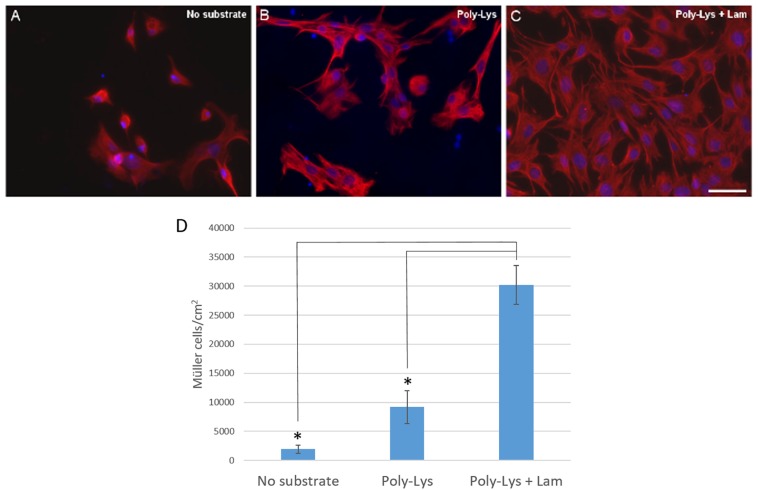
Analysis of the culture of pig Müller cells on different substrates: uncoated coverslips, poly-Lys (poly-L-lysine) and poly-Lys + Lam (laminin). Images of Müller cells growing on different substrates: uncoated coverslips **(A)**, Poly-Lys **(B)** and Poly-Lys + Lam **(C)**. Müller cells were labeled with an antibody against vimentin (red) and the nuclei were stained with DAPI (blue). Using poly-lysine and laminin as a substrate the Müller cell number increased significantly compared to the cells obtained on poly-L-lysine alone or when the cells were cultured on uncoated coverslips, as represented in the histogram **(D)**: ^∗^*p*-value < 0.05. Scale bar, 50 μm.

The effect of plating the cells on different substrates was also analyzed to select that which favored the survival and proliferation of pig Müller glia. Using a combination of poly-L-lysine and laminin as a substrate, the Müller cells obtained after 7 DIV was (30,166.96 ± 6,697.33 Müller cells/cm^2^) as opposed to that obtained when poly-L-lysine alone was used as the substrate (9,177.25 ± 5,592.40 Müller cells/cm^2^) or when the cells were grown directly on the glass coverslips (1,968.36 ± 1,199.96 Müller cells/cm^2^: Figure 2).

### Culture Media

**FIGURE 3:**
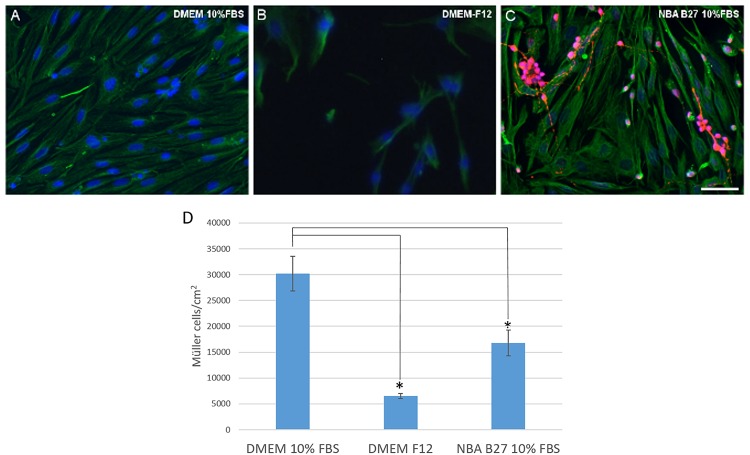
Analysis of pig Müller cells when cultured in different media: DMEM + 10% FBS, DMEM-F12, and NBA/B27 + 10% FBS. The purity and survival of the cells maintained in DMEM + 10% FBS **(A)**, DMEM/F12 **(B)** or NBA B27 + 10% FBS **(C)** was analyzed. Neurons (RGCs) were labeled with an antibody against βIII-tubulin (red), Müller cells with an antibody against vimentin (green) and the nuclei were stained with DAPI. Note that the cultures maintained in NBA/B27 + 10% FBS were not pure **(C)**. In DMEM + 10% FBS, Müller cells reached confluency more rapidly **(A)** and there were fewer Müller cells in the cultures grown in NBA + 10% FBS and DMEM-F12 at both time points, as seen in the histogram **(D)**: ^∗^*p*-value < 0.05. Scale bar, 50 μm.

The purity of the cultures and the number of Müller cells was analyzed when the cells were maintained in three different media: (1) DMEM + 10% FBS; (2) DMEM-F12; and (3) NBA/B27 + 10% FBS. As a result, pig Müller cells reached confluence after 7 DIV in DMEM + 10% FBS (30,233.67 ± 6,697.33 Müller cells/cm^2^), whereas at the same time fewer cells were found when they were grown in DMEM-F12 medium (6,552.97 ± 964.21 Müller cells/cm^2^). When cultured in NBA/B27 + 10% FBS the number of Müller cells obtained was still significantly lower (16,759.31 ± 4,946.93 Müller cells/cm^2^) and the cultures were not pure, since a significant number of neurons were evident, mainly RGCs (Figure 3).

### Cell Culture Characterization

**FIGURE 4:**
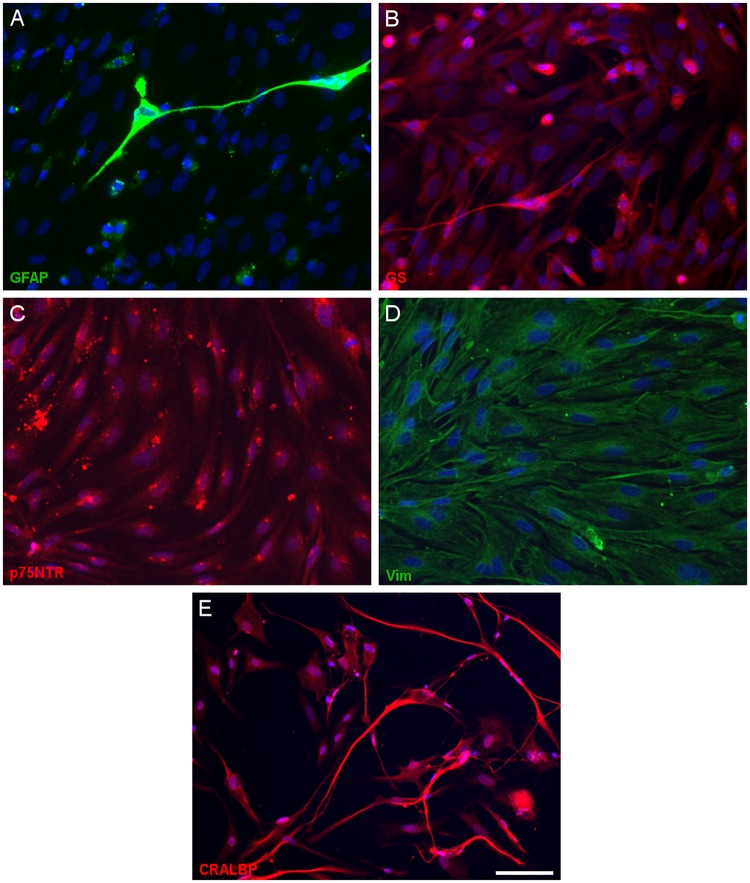
Expression of Müller cell markers in adult pig Müller cell cultures. Images of Müller cells labeled with antibodies raised against GFAP (green, **A**), glutamine synthetase (GS, red, **B**), p75NTR (red, **C**), vimentin (green, **D**) and CRALBP (red, **E**): Scale bar, 50 μm.

**FIGURE 5:**
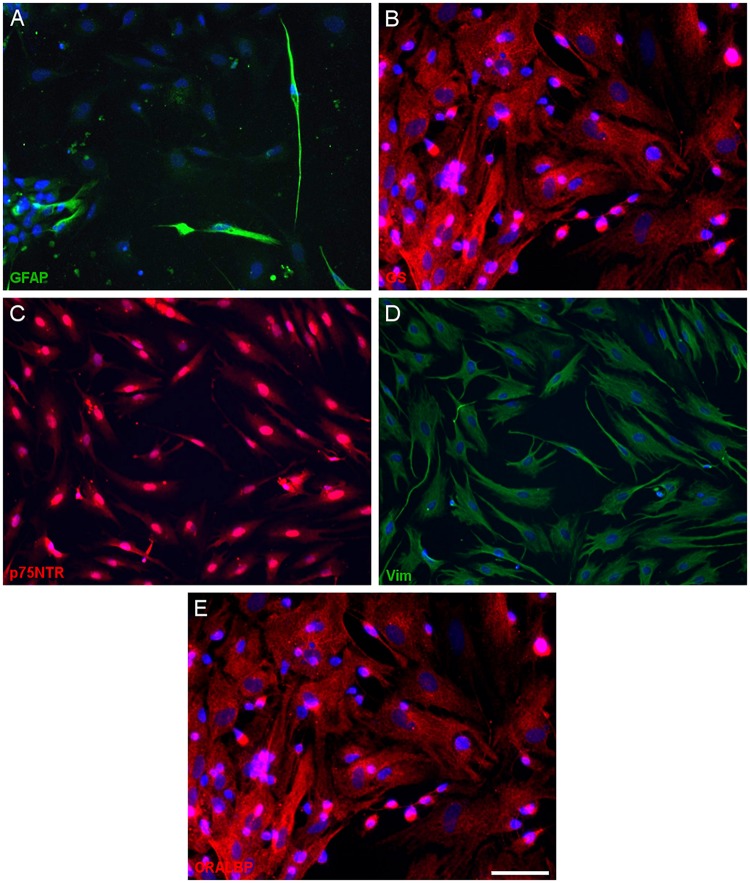
Expression of Müller cell markers in adult rat Müller cell cultures. Images of Müller cells labeled with antibodies against GFAP (green, **A**), glutamine synthetase (GS, red, **B**), p75NTR (red, **C**), vimentin (green, **D**) and CRALBP (red, **E**). Scale bar, 50 μm.

**FIGURE 6:**
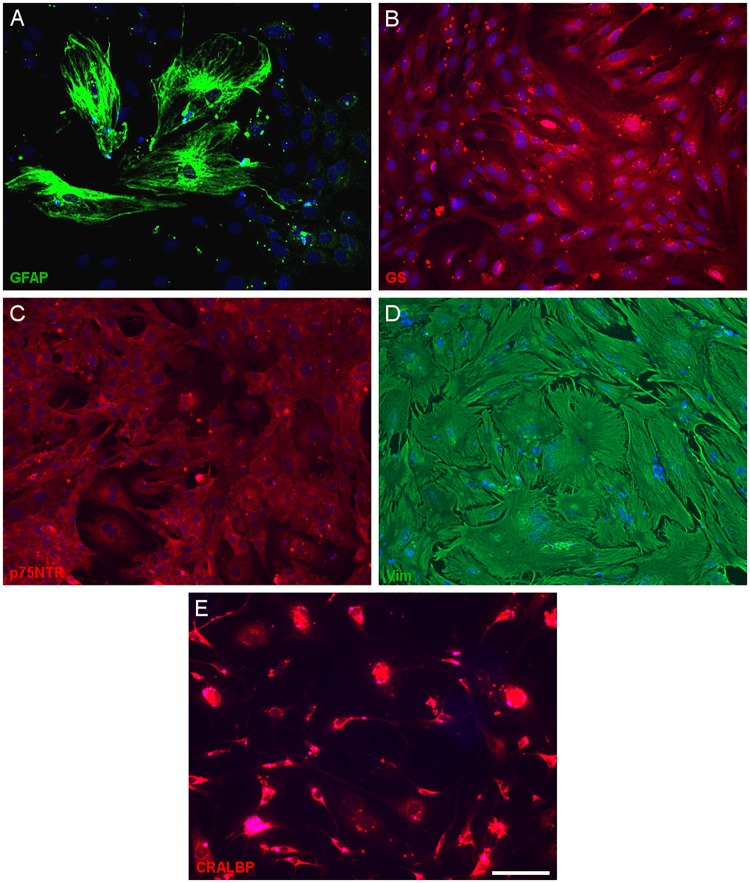
Expression of Müller cell markers in adult mouse Müller cell cultures. Images from Müller cells labeled with antibodies against GFAP (green, **A**), glutamine synthetase (GS, red, **B**), p75NTR (red, **C**), vimentin (green, **D**) and CRALBP (red, **E**). Scale bar, 50 μm.

Having defined the optimal culture conditions using the pig retina, the expression of molecular markers of Müller cells was analyzed in the cultures of adult pig, rat and mouse retinas, specifically the expression of glutamine synthetase (GS), glial fibrillary acidic protein (GFAP), p75NTR, Vimentin, and CRALBP (Figures 4–6). The expression of the specific Müller glia markers was evident in each of the different animals after 7 DIV. The negative control for each secondary antibody employed was also performed in order to verify the primary antibodies labeling (Supplementary Figure S1).

**FIGURE 7:**
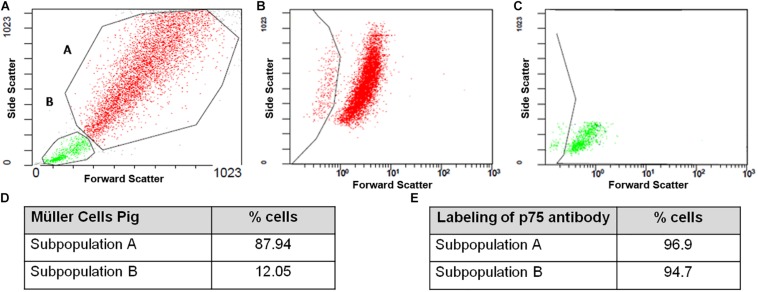
Flow cytometry analysis of pig Müller cell cultures at 7 DIV. Note that two subpopulations of cells were identified based on their physical properties, A and B **(A–C)**, with different proportions of cells in each subpopulation **(D)**. Both the A and B subpopulations express p75NTR **(E)**.

Once the purity of the cultures was assessed by immunocytochemistry the physical properties of the cells in culture were analyzed by flow cytometry. At 7 DIV, two subpopulations of Müller cells were evident (A and B) that were discriminated by their physical properties, since forward scatter can discriminate cells by size while the side scatter measurement provides information about their internal complexity (e.g., granularity: Figures 7A,D). Using an antibody against p75NTR as the only marker in the surface of Müller cells, a 96.9 and 94.7% of the cells in subpopulations A (Figure 7B) and B (Figure 7C) were labeled for p75NTR, respectively (Figure 7E).

**FIGURE 8:**
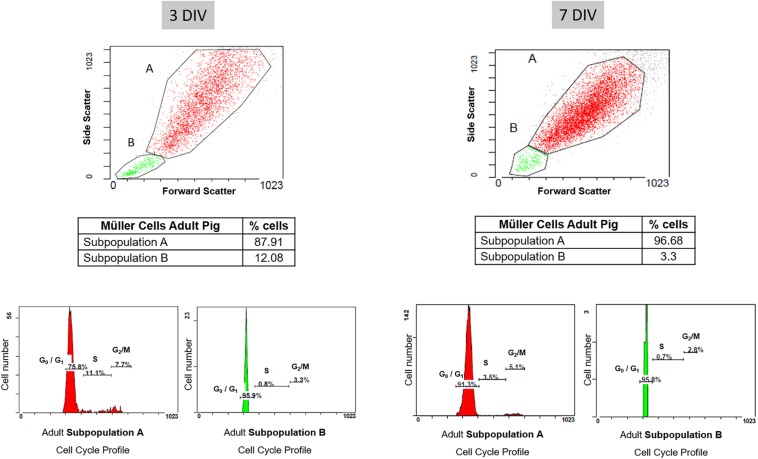
Cell cycle profile of pig Müller cells after 3 and 7 days in culture. The graphs illustrate the proportion of each subpopulation of Müller cells in the different phases of the cell cycle, reflected by the intensity of propidium iodide. Note that once the cells reached confluency (7 DIV), the cell cycle profile indicated there were fewer cells in S phase and G2/M in both subpopulations relative to the cultures at 3 DIV.

In addition, cell cycle FACS profiles of porcine Müller cell cultures were assessed after 3 and 7 DIV. The graphs illustrate the proportions of the cells in the different phases of the cell cycle, which correlates to the propidium iodide intensity. After 3 DIV, the cell cycle profile of the subpopulation A indicated that 75.8% of the cells were in G0/G1, 11.1% in S phase and 7.7% in G2/M. In subpopulation B, 95.9% of the cells were in G0/G1, 0.8% in S phase and 3.3% in G2/M at that time point. The cell cycle profile at 7 DIV showed that 91.3% of the cells in subpopulation A were in G0/G1, 3.5% in S phase and 5.1% in G2/M. Similarly, 95.8% of the cells in subpopulation B were in G0/G1, 0.7% in S phase and 2.8% in G2/M (Figure 8 and Table 3).

**TABLE 3:** Results from the cell cycle profile analysis of porcine Müller cell cultures.

	3 DIV			7 DIV		
	Total			Total			
A	87.91%			96.68%			
	G0/G1	S	G2/M	G0/G1	S	G2/M
	75.8	11.1	7.7	91.3	3,5	5.1

	**Total**			**Total**		
**B**	**12%**			**3.3%**		
	**G0/G1**	**S**	**G2/M**	**G0/G1**	**S**	**G2/M**

	95.9	0.8	3.3	95.8	0.7	2.8

*A, subpopulation A; B, subpopulation B; DIV, Days *in vitro*.*

**FIGURE 9:**
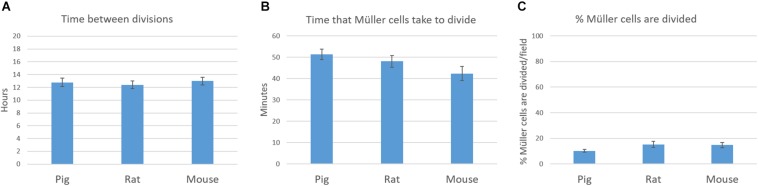
Time-lapse analysis of the proliferation kinetics of pig, rat and mouse Müller cell cultures. The time between divisions **(A)**, the time that Müller cells take to divide **(B)** and the number of Müller cells that are dividing per field **(C)** were quantified and represented in graphs. No significant differences were observed between pig, rat and mouse Müller cell cultures: *p*-value > 0.05.

The behavior of the Müller cells in culture was also analyzed using time-lapse video. In this time-lapse analysis of the proliferation kinetics of cultured pig, rat and mouse Müller cells, the time between divisions, the time that Müller cells take to divide and the number of Müller cells that divided per field was quantified. The time between divisions of Müller cells was 12.77 ± 3.14, 12.40 ± 2.78, and 13 ± 2.89 h for pig, rat and mouse cultures, respectively. Regarding the time that Müller cells take to divide, this was quantified as 51.42 ± 11.08, 48.09 ± 12.49, and 42.38 ± 15.13 min in pig, rat and mouse cultures, respectively. Finally, the number of Müller cells that divided per field was analyzed every 8 h, considering this to be the shortest time found between Müller cells divisions. The percentage of Müller cells that divided was 10.17 ± 3.22% for pig, 15.25 ± 7.9% for rat and 14.76 ± 5.75% for mouse Müller cells. Significant differences were not observed between these pig, rat and mouse Müller cell cultures (Figure 9) and a sample time-lapse video of an adult pig Müller cell culture is shown (Supplementary Video S1).

## Discussion

We describe here a detailed protocol for the easy and reproducible isolation and culture of adult Müller cells. To find the best method to culture Müller cells, we systematically tested different elements in the protocol: the enzymes for digestion of the tissue, the substrates, and the culture medium. To date, the methods described in the literature focus on the isolation of the Müller cells from neonatal or very young animals. Here we focused our attention on obtaining pure primary adult Müller cell cultures, which we consider to be a more useful tool to study degenerative retinal diseases.

To determine the best method to culture adult Müller cells, we first assessed two different enzymes to digest the retinal tissue. As papain and trypsin have been commonly used in previous protocols (Table 1), both were tested over the same period of incubation. More Müller cells were obtained when retinas were digested with papain and these cultures reached confluence at 7 DIV. The cultures derived from trypsin digested retinas take longer to reach confluence, probably due to the lower number of cells that survived the digestion. Thus, we conclude that papain is a gentler enzyme for the digestion of the retinas than trypsin, consistent with reports that trypsin might be more toxic to central neurons than papain (Tabata et al., 2000).

Regarding the substrate for Müller cells, we cultured the cells on uncoated glass coverslips, or on coverslips coated with poly-L-lysine alone or in conjunction with laminin. As expected, the survival and proliferation of Müller cells was more optimal when they were grown on poly-L-lysine + laminin. Poly-L-lysine was tested as it is the most common substrate used in previous protocols (Table 1). Indeed, poly-L-lysine improves cell adherence due to the interaction between the positively charged polymer and negatively charged cells or proteins (Mazia et al., 1975). These data were consistent with our previous studies showing that poly-L-lysine and laminin is the best substrate for retinal cells, including Müller cells (Garcia et al., 2002; Ruzafa and Vecino, 2015; Vecino et al., 2015). Laminin exerts a variety of biological activities, not only mediating cell attachment but also, influencing cell proliferation, differentiation and motility (Paulsson, 1992). Indeed, the end-feet of Müller glial at the inner limiting membrane were found to be enriched in laminin-1 *in vivo* and *in vitro*, and laminin-1 promotes the motility of Müller glial cells (Mehes et al., 2002; Vecino and Kwok, 2016).

A crucial step in cell culture is the selection of the appropriate *in vitro* growth medium. A typical culture medium is composed of a complement of amino acids, vitamins, inorganic salts, glucose, and serum as a source of growth factors, hormones and attachment factors. Here we selected three different culture media based on those most commonly used in previous protocols: DMEM + 10% FBS; DMEM-F12; and NBA B27 + 10% FBS. DMEM-F12 is an extremely rich and complex medium that was designed to contain nutrients, growth factors and hormones instead of requiring a serum supplement (Mather et al., 1981). However, the number of Müller cells in the cultures at 7 DIV was significantly lower in this medium than when the cells were grown in DMEM + 10% FBS. It is known that the secretome of primary Müller cells in culture is influenced by the culture conditions (Ruzafa et al., 2018). Indeed, Müller cell proliferation is stimulated by numerous growth factors and cytokines derived from blood serum (Ikeda and Puro, 1994), which might explain the differences observed when serum-free DMEM-F12 was used as the medium. In the third media tested, NBA B27 + 10% FBS, pure cultures were not obtained due to the growth of neurons in the culture. In fact, Neurobasal-A is a basal medium that is designed to meet the specific requirements of neuronal cells in culture. Thus, with this supplemented NBA medium we developed co-cultures that in addition can be very useful for other applications (Pereiro et al., 2018). In conclusion, it is clear of the media tested that DMEM + 10% FBS is the best option to obtain pure Müller cell cultures as this combination limits the appearance of neurons in the culture while encouraging the proliferation of Müller cells.

In order to confirm that Müller cells retain their characteristics after 7 DIV, we analyzed the expression of known Müller cell markers in pig, rat and mouse cultures: GFAP (Lewis et al., 1988), GS (Mack et al., 1998), p75NTR (Schatteman et al., 1988), and Vimentin (Davidson et al., 1990). We confirmed the expression of these molecular markers in our cultures of adult mammalian Müller cells, validating our protocol. However, while the presence of other glial cells like astrocytes cannot be completely ruled out, there were very few small DAPI stained nuclei in the cultures and nor was there any specific Iba1 staining of microglia. Some slight differences have been shown regarding the morphology of Müller cells between species. However, these variations could be due to the own characteristics of these species and their different behavior in culture (House et al., 1998).

Once the purity of adult pig Müller cell cultures was confirmed by immunocytochemical analysis, Müller cell population was characterized by flow cytometry. This easy, quick, robust and reliable technology serves to classify cell populations according to size, cytoplasmic complexity and the differential expression of surface, cytoplasmic and nuclear markers. Here we used the p75NTR as a membrane marker detected by specific staining with fluorochrome-conjugated reagents (Othmer and Zepp, 1992). Accordingly, two subpopulations of Müller cells were detected that were distinguished by their physical properties (A and B), although population (B) only represented 12.05% of the total Müller cell population on the 3^rd^ DIV and 3.3% on the 7th DIV. These cells might correspond to cells that have just divided as they are smaller and less complex than the cells in subpopulation A, and most of them are in the G0/G1 phase of the cell cycle at both 3 and 7 DIV. The heterogeneity of glial cells is widely known (Luna et al., 2010; Vecino et al., 2016) and hence, not all Müller cells in a retina may respond to a pathogenic stimulus in the same way. Indeed, these cells even have heterogeneous expression of proteins like GFAP, possibly due to the distinct roles they fulfill in the retina (Bringmann et al., 2006). It is known that in the first days of the culture Müller cells increase the expression of GFAP diminishing the expression as culture progresses (Guidry, 1996); however, there are some Müller cells that maintain de expression of GFAP, unlike the homogeneity of the Müller cells for other markers as vimentin. Hence, it is not surprising to find different subpopulations of Müller cells distinguished on the basis of their physical properties. It is noteworthy that the proportion of the A subpopulation of Müller cells in G0/G1 increases at 7 DIV, and the proportion of Müller cells in S phase and G2/M was reduced. This fact suggests that by day 7 the Müller cell culture is static, as it has reached confluence, with only a small proportion of dividing cells.

It is important to note that there were no significant differences in the pig, rat, and mouse cultures of Müller cells in terms of proliferation kinetics, including the time between divisions, the time that Müller cells take to divide and the number of Müller cells that are dividing in each field. As such, it appears that our protocol for Müller cell culture is reproducible in different mammalian species. In addition, the analysis of the Müller glia cell profile obtained from pig retinas, 11.1% of cells in S phase and 7.7 in G2/M phase, are consistent with the time-lapse results obtained, in which 10.17% of Müller cells were dividing per field in an 8 h timeframe. Moreover, the cell cycle profile of adult pig Müller cell cultures adopted the typical characteristics of cells from control primary cell cultures (Pozarowski and Darzynkiewicz, 2004).

The establishment of this Müller cell culture protocol from adult tissues tested in different species could improve some aspects of the studies performed with Müller cell lines. One of the advantages of cell lines was that cells divided rapidly; however, we get confluence in a short period of time (7 DIV). Besides, considering that some characteristic features of primary Müller cells may be altered or lost after 14 DIV in culture (Hauck et al., 2003; Merl et al., 2012) and the phenotype of cell lines is far away from the *in vivo* retina, our protocol allows a period of time in which Müller cells are as closely as possible to *in vivo* condition.

**FIGURE 10:**
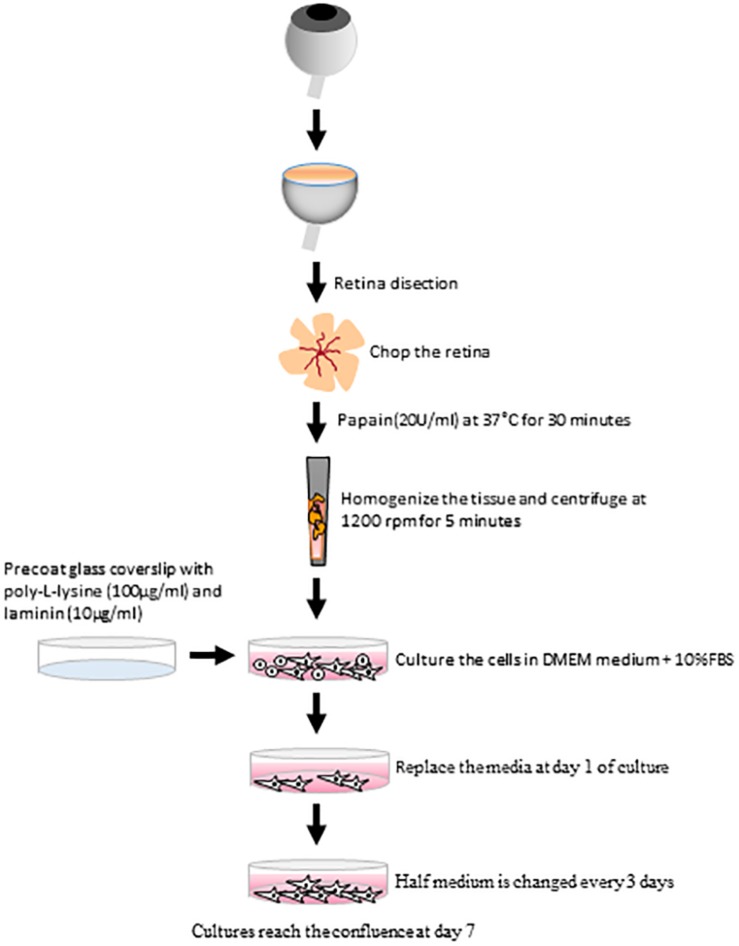
Scheme summarizing the main steps of the protocol to establish adult Müller cell cultures. The culture method for pig Müller cells from adult animals uses papain as the enzyme to digest the retina, poly-L-Lysine and laminin as the substrate and DMEM + 10% FBS as the culture medium.

In summary, we present here a reliable method to obtain and culture mammalian Müller cells from adult retinas, using papain as the enzyme to digest the tissue, poly-L-Lysine and laminin as the substrate, and DMEM + 10% FBS as the culture medium (Figure 10). Compared to other published protocols, that presented here yields more cells and it is less time-consuming. The protocol can easily be adapted to other mammalian species, as seen in rats and mice. As such, this method will help test the effects of drugs on adult Müller cells, assisting researchers in their efforts to study therapeutic targets for retinal diseases like glaucoma, diabetic retinopathy, age-related macular degeneration and retinitis pigmentosa.

## Data Availability Statement

The datasets generated for this study are available on request to the corresponding author.

## Ethics Statement

The animal study was reviewed and approved by the CEEA Vecino/M20/2016/203.

## Author Contributions

XP and EV conceived and designed the experiments and contributed in the data analysis. NR contributed in the imaging tools. XP and EV wrote the manuscript. XP, EV, NR, AA, and AU contributed to manuscript revision and read, and approved the submitted version.

## Conflict of Interest

The authors declare that the research was conducted in the absence of any commercial or financial relationships that could be construed as a potential conflict of interest.

## Supplementary Material

FIGURE S1 Secondary antibody controls on Müller cell cultures. Goat anti-rabbit and mouse secondary antibodies Alexa Fluor 488 controls in pig **(A)**, rat **(C)** and mouse **(E)** Müller cell cultures and goat anti-rabbit and mouse secondary antibodies Alexa Fluor 555 controls in pig **(B)**, rat **(D)**, and mouse **(F)** Müller cells cultures. Scale bar, 50 μm.

VIDEO S1 Time-lapse video of an adult pig Müller cell culture.
