# Supplementary figures and images for: Optimization of a Method to Isolate and Culture Adult Porcine, Rats and Mice Müller Glia in Order to Study Retinal Diseases
Source: Front Cell Neurosci. 2020 Jan 29;14:7. doi: 10.3389/fncel.2020.00007 (PMC7004099; doi:10.3389/fncel.2020.00007)

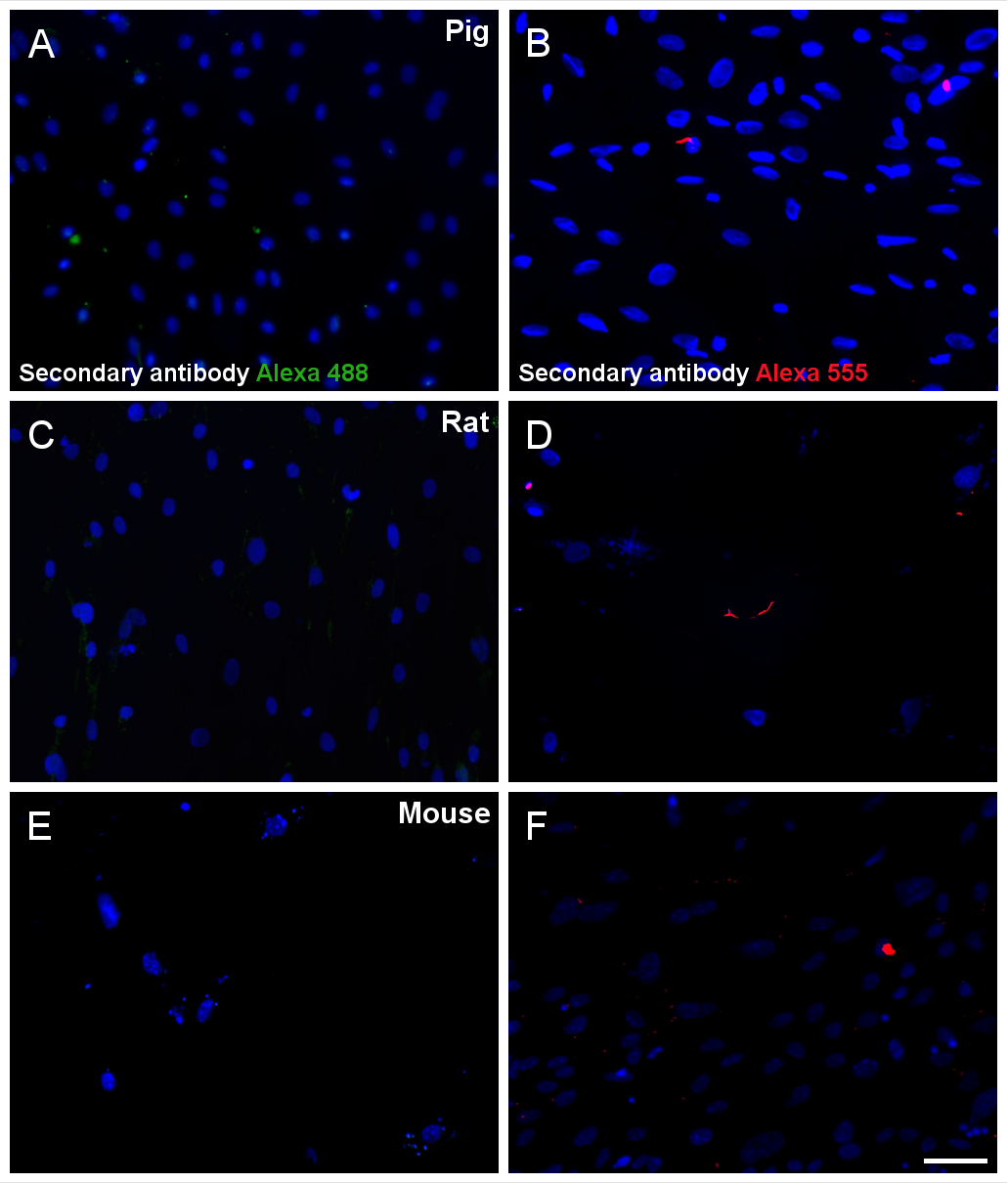

Supplement: FIGURE S1 — Secondary antibody controls on Müller cell cultures. Goat anti-rabbit and mouse secondary antibodies Alexa Fluor 488 controls in pig (A), rat (C) and mouse (E) Müller cell cultures and goat anti-rabbit and mouse secondary antibodies Alexa Fluor 555 controls in pig (B), rat (D), and mouse (F) Müller cells cultures. Scale bar, 50 μm. [file Image_1.TIFF]
